# Non‐coordinated and Hydrogen Bonded Phenolate Anions as One‐Electron Reducing Agents

**DOI:** 10.1002/chem.202005123

**Published:** 2021-02-08

**Authors:** Robin F. Weitkamp, Beate Neumann, Hans‐Georg Stammler, Berthold Hoge

**Affiliations:** ^1^ Centrum für Molekulare Materialien Fakultät für Chemie Universität Bielefeld Universitätsstraße 25 33615 Bielefeld Germany

**Keywords:** hydrogen bond, phenol, phenolate, radical anion, SF_6_ activation

## Abstract

In this work, the syntheses of non‐coordinated electron‐rich phenolate anions via deprotonation of the corresponding alcohols with an extremely powerful perethyl tetraphosphazene base (Schwesinger base) are reported. The application of uncharged phosphazenes renders the selective preparation of anionic phenol‐phenolate and phenolate hydrates possible, which allows for the investigation of hydrogen bonding in these species. Hydrogen bonding brings about decreased redox potentials relative to the corresponding non‐coordinated phenolate anions. The latter show redox potentials of up to −0.72(1) V vs. SCE, which is comparable to that of zinc metal, thus qualifying their application as organic zinc mimics. We utilized phenolates as reducing agents for the generation of radical anions in addition to the corresponding phenoxyl radicals. A tetracyanoethylene radical anion salt was synthesized and fully characterized as a representative example. We also present the activation of sulfur hexafluoride (SF_6_) with phenolates in a SET reaction, in which the nature of the respective phenolate determines whether simple fluorides or pentafluorosulfanide ([SF_5_]^−^) salts are formed.

## Graphical abstract

**Effect of hydrogen bonding on redox properties**: The deprotonation of phenol derivatives with a tetraphosphazene base delivers non‐coordinated phenolates with zinc‐like redox potentials of up to −0.72(1) V vs. SCE, which are capable in radical anion synthesis and SF_6_ activation. The addition of phenol or water allows for the selective formation of phenolate adducts and enables the study of the effect of hydrogen bonding on phenolate redox properties.

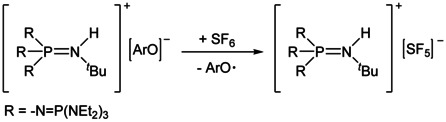

## Introduction

Phenol and phenolates are key compounds in applied chemistry, as documented by the industrial Kolbe–Schmitt process.^[1]^ Moreover, a variety of fundamental reactions within the biosphere, such as the photosynthesis, are strongly related to phenolic functionalities.[^2^, ^3^]

Phenol represents the simplest aromatic alcohol with a pronounced tendency for hydrogen bond formation, which strongly governs the acidity of the present OH functions.^[4]^ Phenol derivatives with a higher acidity than phenol are deprotonated by tetraalkylammonium hydroxides, yielding the corresponding ammonium phenolates.^[5]^ Interestingly, as reported by Reetz et al., all attempts to isolate the non‐coordinated phenolate [H_5_C_6_‐O]^−^ ([PhO]^−^) anion by deprotonation with tetra‐*n*‐butylammonium hydroxide invariantly led to a phenol‐phenolate adduct featuring a moderately strong hydrogen bond (O−O distance of 247.1(5) pm).^[6]^ Pronounced hydrogen bonding is also present in imidazolium phenolates, which feature strong C−H⋅⋅⋅O^−^ cation‐anion interactions.[^7^, ^8^]

The investigation of hydrogen bonding in proton‐coupled electron transfer processes is of growing interest, particularly because of its relevance towards the photosystem.[^3^, ^9^] The high basicity of the tetraphosphazene base [(Et_2_N)_3_P=N]_3_P=N*t*Bu (**1**) is sufficient for the deprotonation of phenol, as discussed previously. ^[10]^ The proton of the corresponding phosphazenium cation [**1H**]^**+**^ is well shielded towards nucleophilic attack, which allows the isolation of salts with non‐coordinated phenolate anions. Thus, in the absence of cation‐anion interactions, the effect of hydrogen bonding on the redox properties of phenolate anions can be investigated in detail. The presence of water also effects the oxidation potential of phenol,^[10]^ which casts doubt on the reported phenolate redox data from the literature, which were obtained from phenolates generated by deprotonation with tetraalkylammonium hydroxide hydrates in acetonitrile solution.[^11^, ^12^] The elucidation of the influence of hydrogen bonding requires uncharged phosphazene bases for the deprotonation of phenols to create a definite design of hydrogen bonded phenol‐phenolate adducts or phenolate hydrates. Here, in contrast to the application of alkylammonium hydroxide hydrates, the degree of hydration can be controlled exactly by the added amount of water to the reaction. Furthermore, hydrogen bonding also strongly influences light absorption and emission of fluorophores.^[13]^ This phenomenon is also observed for 2‐naphtholate anions,^[14]^ and the fluorescence of 2‐naphtholate was investigated in more detail in the presence of imidazolium‐based ionic liquids, which are able to form C−H⋅⋅⋅O^−^ hydrogen bonds.[^8^, ^15^] Therefore it is obvious to investigate light absorption and emission of the non‐coordinated 2‐naphtholate anion in comparison to its free 2‐naphthole and its adduct with the anion.

**Scheme 1:**
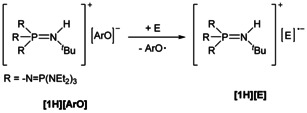
Application of phosphazenium phenolates as reducing agent.

Phenolate anions possess a pronounced tendency for single‐electron transfer (SET) reactions, as the resulting phenoxyl radicals are well stabilized by electron delocalization. Obviously, we are interested in testing phosphazenium phenolates as electron donors in SET processes. As depicted in Scheme 1, neutral electrophiles are reduced under liberation of stable phenoxyl radicals, which are reluctant to further reactions, and by the generation of the corresponding phosphazenium salts of reactive radical anions [E]^.−^.

The applied phenolate should fulfil several prerequisites as a high electron density leading to a sufficiently negative redox potential. Bulky substituents in 2, 4 and 6 position are necessary for the stabilization of phenoxyl radicals by mitigating its nucleophilicity and by obstructing their dimerization.^[12]^ Consistently, we selected 2,6‐di‐*tert*‐butyl substituted phenolates as the substrates of choice.

## Results and Discussion

### Syntheses of non‐coordinated phenolate anions

**Scheme 2:**
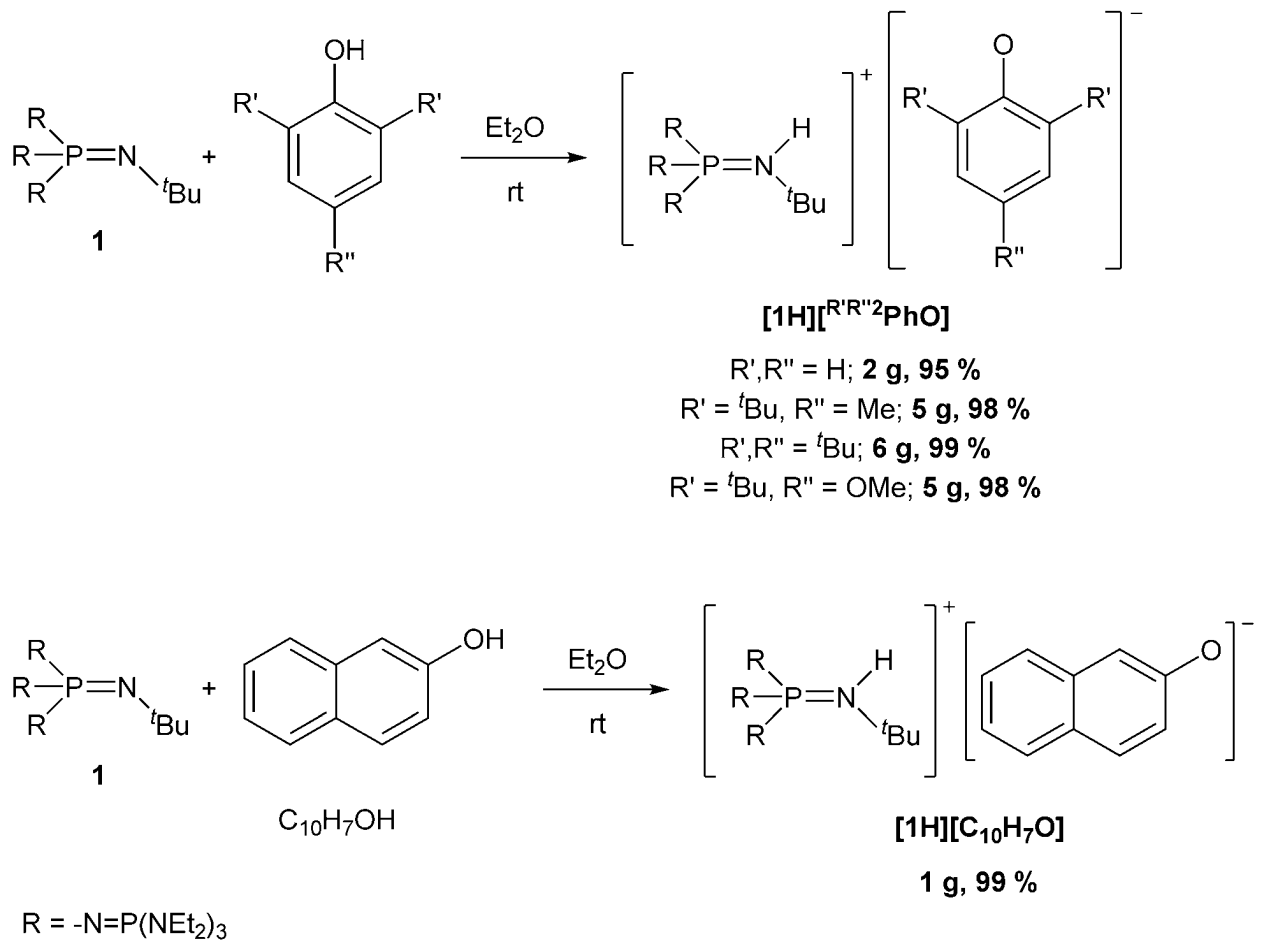
Synthesis of non‐coordinated phenolate salts using **1**.

The perethyl tetraphosphazene base **1** was synthesized on a multigram scale according to the procedure described previously.^[16]^ The reaction of **1** with phenols in ethereal solution affords the corresponding salts as microcrystalline solids in excellent yields (>95 %, Scheme 2). Importantly, the products are devoid of significant cation‐anion contacts.

**Figure 1:**
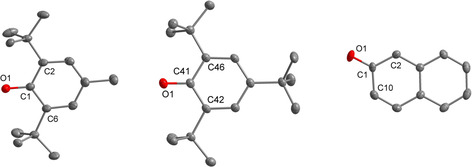
Molecular structures of non‐coordinated phenolate anions in **[1H][^Me*t*Bu2^PhO]** (left), **[1H][** ^***t*****Bu3**^ **PhO]** (middle) and **[1H][C_10_H_7_O]** (right). Thermal ellipsoids are shown at 50 % probability. The hydrogen atoms bonded at carbon atoms are omitted for clarity. Selected bond lengths [pm]: left: C1−O1 128.5(2), C1−C2 144.9(2), C1−C6 144.8(2); middle: C41−O1 129.8(2), C41−C42 144.3(2), C41−C46 144.8(2); right: C1−O1 128.4(2), C1−C2 142.1(2), C1−C10 145.8(2).

Whereas salts **[1H][PhO]**, **[1H][^Me*t*Bu2^PhO]** and **[1H][** ^***t*****Bu3**^ **PhO]** are colorless, salt **[1H][^MeO*t*Bu2^PhO]** shows a deep yellow color. The deprotonation of 2‐naphthol (C_10_H_7_OH, Scheme 2) afforded the saline naphtholate **[1H][C_10_H_7_O]** with the non‐coordinated anions in nearly quantitative yield as fluorescent green crystals. All compounds are air sensitive and by oxidation change their color to yellow, purple, brown or rust‐red, while the color of **[1H][C_10_H_7_O]** quickly fades. The salts deteriorate in Brønsted acids and solvents like chloroform, dichloromethane and acetonitrile. Thus, handling these phenolates in THF or ethereal solution is indispensable. The novel phenolates were fully characterized and molecular structures were elucidated by single‐crystal X‐ray diffraction (Figure 1) using crystals collected from the cooled ethereal reaction mixtures.

As discussed previously,^[10]^ salt **[1H][PhO]** exhibits the first non‐coordinated phenolate [H_5_C_6_‐O]^−^ anion as building block. The ^13^C NMR resonance of the C‐O^−^ carbon atom in [PhO]^−^ (*δ*=175.0 ppm) is significantly deshielded in comparison to the anions [C_10_H_7_O]^−^ (*δ*=173.7 ppm), [^Me*t*Bu2^PhO]^−^ (*δ*=170.2 ppm), [^*t*Bu3^PhO]^−^ (*δ*=170.3 ppm) and [^MeO*t*Bu2^PhO]^−[10]^ (*δ*=168.0 ppm). The corresponding C‐O^−^ distances of these substituted phenolates do not differ significantly from that of [PhO]^−^ (128.7(2) pm, Table 1), but are shortened compared to coordinated anions as in NaOPh (133(1) pm).^[17]^

**Table 1:** Selected bond lengths, angles and redox potentials (E^0^) of non‐coordinated and hydrogen bonded phenolate anions with **[1H]^+^** as the counterion. For disordered molecules the values of the major representatives are depicted.

Anion	Bond	Distance [pm]	Angle [°]^[a]^	*E* ^0^ [V]^[b]^
[PhO]^−^	C−O^−^	128.7(2)	115.0(1)	−0.12(1)^[c]^
[^Me*t*Bu2^PhO]^−^	C−O^−^	128.5(2)	116.5(2)	−0.62(1)
[^*t*Bu3^PhO]^−^	C−O^−^	129.8(2)	116.4(1)	−0.52(1)
[^MeO*t*Bu2^PhO]^−^	C−O^−^	129.0(2)	116.8(3)	−0.72(1)
[C_10_H_7_O]^−^	C−O^−^	128.4(2)	114.3(1)	−0.15(1)^[c]^
[PhO(H_2_O)]^−^	C−O^−^ O(H)−O^−^	129.8(1) 260.8(7) 265.2(7)	115.4(8)	−0.04(1)^[c]^
[(PhO)_2_H]^−^	C−O^−^ O(H)−O^−^	131.9(2) 132.1(2) 243.7(2)	117.2(1) 118.0(1)	+0.22(1)^[c]^
[(^MeO*t*Bu2^PhO)_2_H]^−^	C−O^−^ O(H)−O^−^	131.3(1) 136.0(1) 247.0(1)	118.0(1) 120.5(1)	−0.70(1)
[(C_10_H_7_O)_2_H]^−^	C−O^−^ O(H)−O^−^	132.1(2) 133.0(6) 238.5(4)	118.1(1) 115.8(3)	+0.08(1)^[c]^

[a] The *ortho*‐*ipso*‐*ortho* carbon atom angle relative to the C‐O^−^ function is depicted. [b] Voltammograms recorded in 0.1 m [NBu_4_][PF_6_]THF solution at 100 mV s^−1^ under inert atmosphere with a glassy carbon working electrode (2.0(1) mm), a counter electrode (steel 18/8, 2.0(1) mm) and an Ag/AgCl reference electrode. All potentials were calibrated to the Fc/Fc^+^ couple (+0.405 V vs. SCE). [c] Due to irreversible redox reaction, only the oxidation potential (*E*_Ox_) is displayed.

For the sake of a complete picture, we additionally tested the deprotonation of ^MeO*t*Bu2^PhOH with tetra‐*n‐*butylammonium hydroxide (triacontahydrate) in a mixture of diethyl ether and THF with a subsequent work‐up. Since the in situ deprotonation leads to the formation of the hydrate **[NBu_4_][^MeO*t*Bu2^PhO(H_2_O)**_***n***_ **]**, we focused on the investigation of a possible liberation of the free anion [^MeO*t*Bu2^PhO]^−^ by drying the hydrate in a high vacuum. The powdery pale yellow solid, which was obtained after removal of all volatiles, shows a signal of the C−O^−^ carbon atom at *δ*=164.1 ppm in the ^13^C NMR spectrum, which is shifted upfield by about 4 ppm compared to **[1H][^MeO*t*Bu2^PhO]** (*δ*=168.0 ppm). In the IR spectrum no OH stretching vibration is observed, which points to the absence of OH groups evoked by phenol or water. Recrystallization of the salt from a diethyl ether/ THF solution at −28 °C afforded single crystals suitable for X‐ray analysis. The investigation shows the free anion in **[NBu_4_][^MeO*t*Bu2^PhO]**, which is not hydrated and does not show any significant contacts to the cation with the shortest C−H⋅⋅⋅O^−^ contact of O1−C47* with 340.6(2) ppm (symmetry code C47* (−1/2+X, 3/2−Y, 1−Z). The C1−O1 distance of 129.3(2) ppm is not different from that in the phosphazenium salt. However, air sensitivity evidenced by a color change from yellow to green is attenuated relative to that of the phosphazenium analogue.

### Syntheses of hydrogen bonded phenolates

**Scheme 3:**
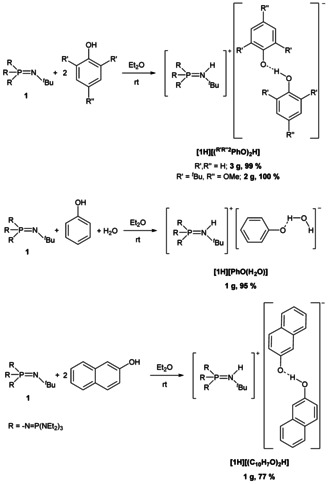
Syntheses of salts featuring anionic hydrogen bonded phenolate adducts by application of phosphazene **1**.

A selective preparation of phenol‐phenolate anions is effected by the deprotonation of phenol by half a molar equivalent of phosphazene **1**, or in case of **[1H][PhO(H_2_O)]** by deprotonation of phenol prior to the addition of one molar equivalent of water (Scheme 3).

Salt **[1H][(PhO)_2_H]** incorporates the anion with an asymmetric, moderately strong hydrogen bond^[18]^ with d(O1‐O8)=243.7(2) pm and therefore the ^13^C NMR resonance of the C‐O^−^ carbon atoms of *δ*=167.2 ppm is shifted upfield in comparison to free [PhO]^−^ (*δ*=175.0 ppm).^[10]^

**Figure 2:**
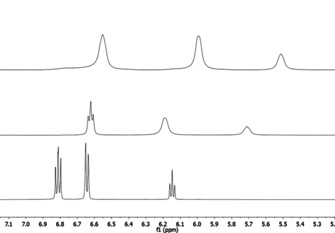
Aromatic region of the ^1^H NMR spectra of **[1H][PhO]** (top), **[1H][PhO(H_2_O)]** (middle) and **[1H][(PhO)_2_H)]** (bottom) in [D_8_]THF.

The monohydrate **[1H][PhO(H_2_O)]** is accessible in excellent yields (95 %). Figure 2 displays the aromatic regions of the ^1^H NMR spectra of [PhO]^−^ and its adducts. Hydrogen bonding of [PhO]^−^ with water and phenol brings about significant lowfield shifts of the signals and an improved resolution of couplings. The latter may be rationalized by a reduced delocalization of the negative charge over the aromatic system, evoked by charge withdrawing hydrogen bonding.

In accordance, the ^13^C NMR resonance of the C−O^−^ carbon atom in [PhO(H_2_O)]^−^ (173.6 ppm) is slightly upfield shifted relative to [PhO]^−^ (175.0 ppm) and shielded relative to [(PhO)_2_H]^−^ (167.2 ppm).

**Figure 3:**
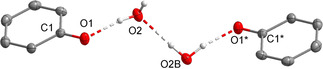
Molecular structure of the formed dianion of salt **[1H][PhO(H_2_O)]**. Thermal ellipsoids are shown at 50 % probability. Hydrogen atoms bonded at carbon atoms and disordered atoms are omitted for clarity. The cation is not shown. The hydrate water molecules are disordered (1:1). Symmetry code of O1* and C1*: (1−X, 1−Y, −Z). Selected bond lengths [pm] and angles [°]: O1−C1 129.8(1), O1−O2 260.8(7), O2−O2B 293.1(5), O1*−O2B 265.2(7); O1‐O2‐O2B 117.0(1), O1*‐O2B‐O2 109.7(2).

Colorless **[1H][PhO(H_2_O)]** deteriorates above 92 °C and thus, is more temperature sensitive than **[1H][PhO]** (dec. >115 °C) with a non‐coordinated [PhO]^−^ anion. The OH vibration modes are detected as a single sharp resonance at 3350 cm^−1^. Single crystals for X‐ray crystallography were grown from the ethereal reaction mixture at −28 °C. In the solid state the phenolate monohydrate anion forms a dimer, in which two phenolate hydrates are associated via hydrogen bonding (Figure 3). Interestingly, the water molecules do not show μ_2_ bridging between two phenolate anions, but are arranged linearly in a zigzag array. The water molecules are disordered in a 1:1 ratio due to symmetry. The hydrogen bonds with O1−O2 and O1*−O2B distances of 260.8(7) pm and 265.2(7) pm, respectively, are significantly elongated compared to phenol‐phenolate hydrogen bonding in **[1H][(PhO)_2_H]** (243.7(2) pm). The O2−O2B distance of 293.1(5) pm is remarkably long.

All attempts to crystallize hemi‐, di‐ or trihydrates of [PhO]^−^ suitable for X‐ray analysis by adding the respective amounts of water to **[1H][PhO]** resulted in the formation of amorphous solids. The investigation of diffracting crystals solely shows the presence of **[1H][PhO(H_2_O)]**.

**Figure 4:**
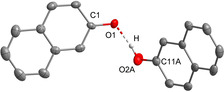
Molecular structure of salt **[1H][(C_10_H_7_O)_2_H]**. Thermal ellipsoids are shown at 50 % probability. Hydrogen atoms bonded at carbon atoms and disordered atoms are omitted for clarity. The cation is not shown. The 2‐naphthol molecule is disordered (70:30). Selected bond lengths [pm]: O1−C1 132.1(2), O2A−C11A 133.0(6), O1−O2A 238.5(4).

The naphthol‐naphtholate salt **[1H][(C_10_H_7_O)_2_H]** is accessible in a 77 % yield (Scheme 3) as a colorless solid with a melting point of 76 °C. Regarding the major representative of salt **[1H][(C_10_H_7_O)_2_H]** (Figure 4), the anion contains the strongest observed hydrogen bond within all phenolate adducts herein with an O−O separation of 238.5(4) pm. The respective OH vibration mode is detected at 3379 cm^−1^, and thus is slightly shifted to higher wavenumbers in comparison to [PhO(H_2_O)]^−^. In the IR spectra of all other hydrogen bonded phenolates this mode is not observed.

**Figure 5:**
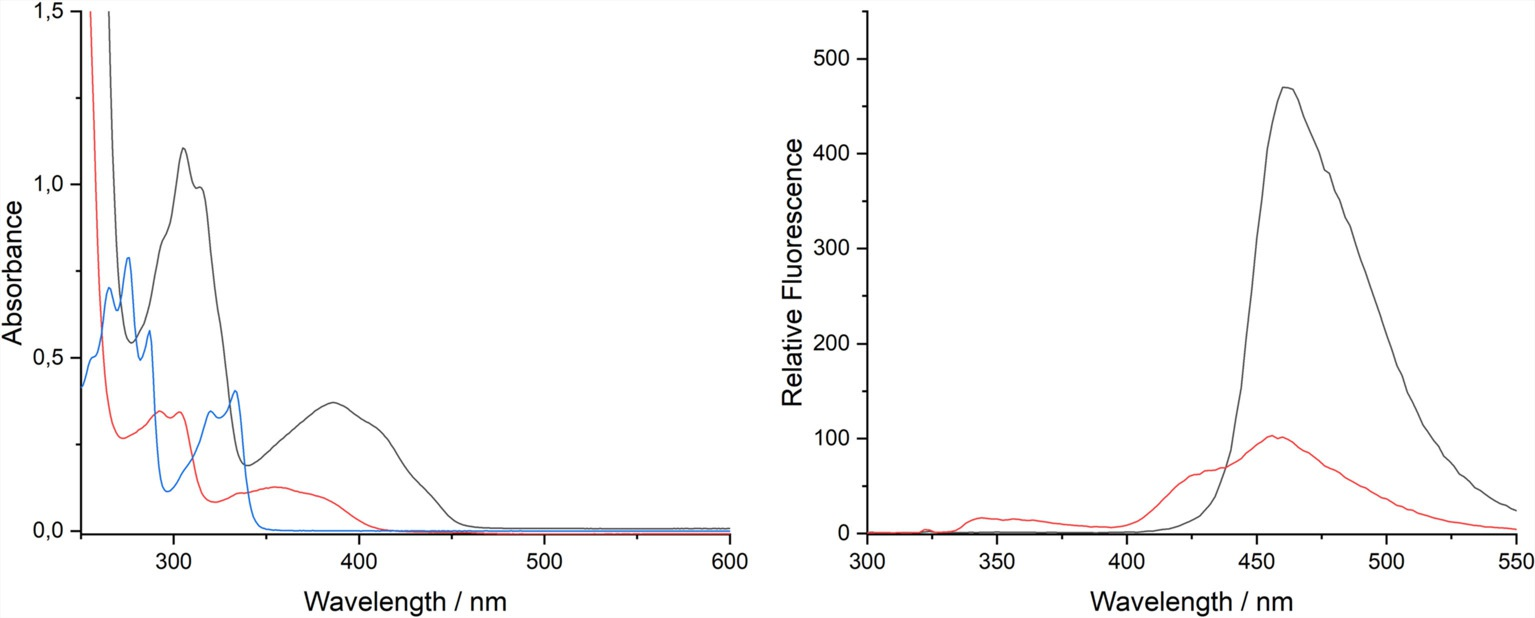
UV/Vis absorption spectrum (left, 32 μm) of 2‐naphthole (C_10_H_7_OH, blue), **[1H][C_10_H_7_O]** (black) and **[1H][(C_10_H_7_O)_2_H]** (red) and fluorescence emission spectra (right, 200 μm, *λ*_ex_=320 nm) of **[1H][C_10_H_7_O]** (black) and **[1H][(C_10_H_7_O)_2_H]** (red).

Interestingly, **[1H][(C_10_H_7_O)_2_H]** containing the hydrogen bonded anion appears colorless, which is in stark contrast to green **[1H][C_10_H_7_O]** featuring the non‐coordinated anion. UV/Vis spectra in dry THF solution clearly reveal the bathochromic absorbance shift of [C_10_H_7_O]^−^ into the visible range with two local maxima at about *λ*=412 nm and 439 nm, while the absorbance of [(C_10_H_7_O)_2_H]^−^ is hypsochromically shifted and observed below 400 nm (Figure 5, left), which agrees with the lack of color. The influence of hydrogen bonding is also visible in fluorescence spectra (Figure 5, right). Fluorescence emission spectra were recorded in dry THF with an excitation wavelength of *λ*_ex_=320 nm. The fluorescence of the non‐coordinated 2‐naphtholate anion in **[1H][C_10_H_7_O]** is of high intensity and displays one strong fluorescence maximum at *λ*_em_=462 nm with a Stokes shift of 9605 cm^−1^. The adduct in [(C_10_H_7_O)_2_H]^−^ displays comparatively low intense fluorescence and exhibits four fluorescence maxima at *λ*_em_=344 nm, 360 nm, 427 nm and 458 nm (Stokes shifts of 2181, 3472, 7831 and 9416 cm^−1^). It is remarkable, that the bands of [C_10_H_7_O]^−^ at *λ*_em_=462 nm and of [(C_10_H_7_O)_2_H]^−^ at *λ*_em_=458 nm are close together, which may suggest a dissociation of the hydrogen bonded adduct in the excited state prior to emission. However, mechanistic insights will be discussed elsewhere.

**Figure 6:**
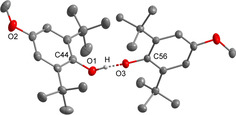
Molecular structure of the anion of salt **[1H][(^MeO*t*Bu2^PhO)_2_H]**. Thermal ellipsoids are shown at 50 % probability. The hydrogen atoms bonded at carbon atoms are omitted for clarity. The cation is not shown. Selected bond lengths [pm]: O1−O3 247.0(1), O1−C44 136.0(1), O3−C56 131.3(2).

Bulky *tert*‐butyl substituents in 2 and 6 positions of phenol do not prevent the formation of hydrogen bonded adducts, as shown for the phenol‐phenolate anion in **[1H][(^MeO*t*Bu2^PhO)_2_H]**, which can be easily obtained in a quantitative yield as a green solid (Scheme 3). However, the hydrogen bond with an O1‐O3 distance of 247.0(1) pm is slightly elongated compared to **[1H][(PhO)_2_H]** (243.7(2) pm), which may result from steric repulsion (Figure 6).

The ^13^C NMR resonance of the C‐O^−^ carbon atoms adjacent to the hydrogen bond are upfield shifted (*δ*=158.5 ppm) compared to free [^MeO*t*Bu2^PhO]^−^ (*δ*=168.0 ppm). Clearly, hydrogen bonding seems responsible for a reduced air‐sensitivity, but increased thermal sensitivity (dec. >74 °C, **[1H][^MeO*t*Bu2^PhO]** dec. >118 °C).

### Cyclic voltammetry of phenolates

**Figure 7:**
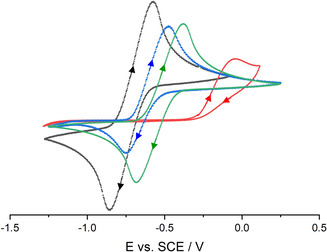
Cyclic voltammograms of non‐coordinated phenolates: **[1H][PhO]** (red), **[1H][** ^***t*****Bu3**^ **PhO]** (green), **[1H][^Me*t*Bu2^PhO]** (blue) and **[1H][^MeO*t*Bu2^PhO]** (black). Voltammograms recorded in 0.1 m [NBu_4_][PF_6_]THF solution at 100 mV s^−1^ under inert atmosphere with a glassy carbon working electrode (2.0(1) mm), a counter electrode (steel 18/8, 2.0(1) mm) and an Ag/AgCl reference electrode. All potentials were calibrated to the Fc/Fc^+^ couple (+0.405 V vs. SCE).

The non‐coordinated and hydrogen bonded phenolates were analyzed by cyclic voltammetry (CV) measurements under inert conditions (Table 1, Figure 7). THF as the solvent and [NBu_4_][PF_6_] as the electrolyte were carefully dried prior to use. The substituted non‐coordinated phenolate anions show the familiar trend of redox values, known from the literature.^[12]^

The determined redox potentials of substituted phenolates vary from *E* ^0^=−0.72(1) V vs. SCE for [^MeO*t*Bu2^PhO]^−^ to −0.52(1) V vs. SCE for [^*t*Bu3^PhO]^−^. These values exceed the reported literature data^[11]^ in acetonitrile solution by about 0.3 V. The anion [^MeO*t*Bu2^PhO]^−^ has the most negative redox potential of the here prepared phenolates and reaches that of zinc, which qualifies the anion as an organic zinc mimic.^[19]^ In contrast to the reversible redox reaction of the sterically encumbered phenolates [^MeO*t*Bu2^PhO]^−^ and [^*t*Bu3^PhO]^−^, the [PhO]^−^ anion shows an irreversible oxidation at *E*_Ox_=−0.12(1) V vs. SCE due to a facile recombination of the formed radicals.^[20]^ The anions [C_10_H_7_O]^−^ and [^Me*t*Bu2^PhO]^−^ are irreversibly oxidized as well.

According to CV measurements, the anion of the salt **[NBu_4_][^MeO*t*Bu2^PhO]** exhibits the same redox potential of E^0^=−0.72(1) V vs. SCE as **[1H][^MeO*t*Bu2^PhO]**. However, the air sensitivity is reduced and the ease of color change upon air contact from yellow to green significantly decreases.

Since the reported potentials of phenolate salts in the literature were preferentially determined in aqueous acetonitrile solution with alkylammonium hydroxide hydrates as the deprotonation agent, the influence of conceivable hydrogen bonding on the obtained potentials is neglected.[^11^, ^12^] The application of **1** enables the investigation with regard to the influence of hydrogen bonding on the oxidation potentials of phenolates.

In keeping with this, we now focused on the cyclic voltammetric investigation of the non‐coordinated phenolate anion [PhO]^−^, as well as of the adducts [PhO(H_2_O)]^−^ and [(PhO)_2_H]^−^. We further looked at the influence of bulky substituents in 2 and 6 positions on the adduct formation and the resulting redox properties.

As described before, the oxidation potential of salt **[1H][PhO]** was determined to *E*_Ox_=−0.12(1) V vs. SCE.^[10]^ This value is significantly cathodically shifted compared to the reported literature data in acetonitrile solution (+0.24 V vs. SCE).^[12]^

**Figure 8:**
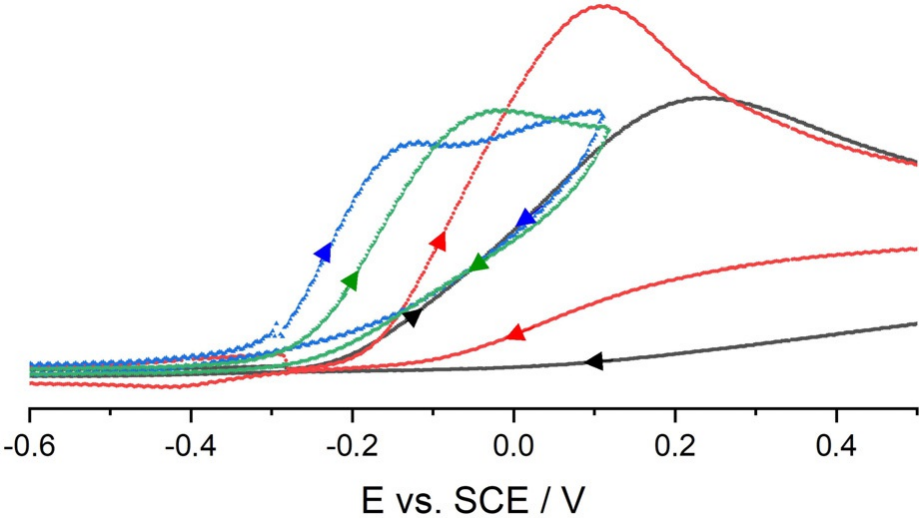
Cyclic voltammograms of **[1H][PhO]** (blue), **[1H][PhO(H_2_O)]** (green), **[1H][(PhO)_2_H]** (black) and **[1H][(PhO)_2_H]**+excess H_2_O (concentration of 0.1 m H_2_O in the electrolyte solution, red). Voltammograms recorded in 0.1 m [NBu_4_][PF_6_]THF solution at 100 mV s^−1^ under inert atmosphere with a glassy carbon working electrode (2.0(1) mm), a counter electrode (steel 18/8, 2.0(1) mm) and an Ag/AgCl reference electrode. All potentials were calibrated to the Fc/Fc^+^ couple (+0.405 V vs. SCE).

Hydrogen bonding to water in the phenolate monohydrate salt **[1H][PhO(H_2_O)]** brings about an anodic shift of *E*_Ox_ to −0.04(1) V vs. SCE (Figure 8). The potential of the anion in **[1H][(PhO)_2_H]** with a value of *E*_Ox_=+0.22(1) V vs. SCE experienced an even stronger anodic shift. This trend is confirmed by calculated ionization potentials at the BP86/6–311+g(3df,2p) level of theory,^[21]^ according to which the non‐coordinated anion [PhO]^−^ (*E*_i_=228.69(1) kJ mol^−1^) is significantly influenced by hydrogen bonding to a water molecule in [PhO(H_2_O)]^−^ (*E*_i_=267.42(1) kJ mol^−1^).^[10]^ The hydrogen bond donation capability in [(PhO)_2_H]^−^ is characterized by a further increase of *E*_i_ to 314.90(1) kJ mol^−1^.

The observations may be rationalized by a reduced charge density on the phenolate oxygen atom, which is affected by the strength of the formed hydrogen bond interaction. Moreover, the strength of the hydrogen bond influences the dissociation of the adduct in solution. Thus, assuming that excessive amounts of water displace phenol in **[1H][(PhO)_2_H]** in the equilibrium reaction, the increased oxidation potential points to the formation of phenolate‐water aggregates (Figure 8).^[10]^

**Figure 9:**
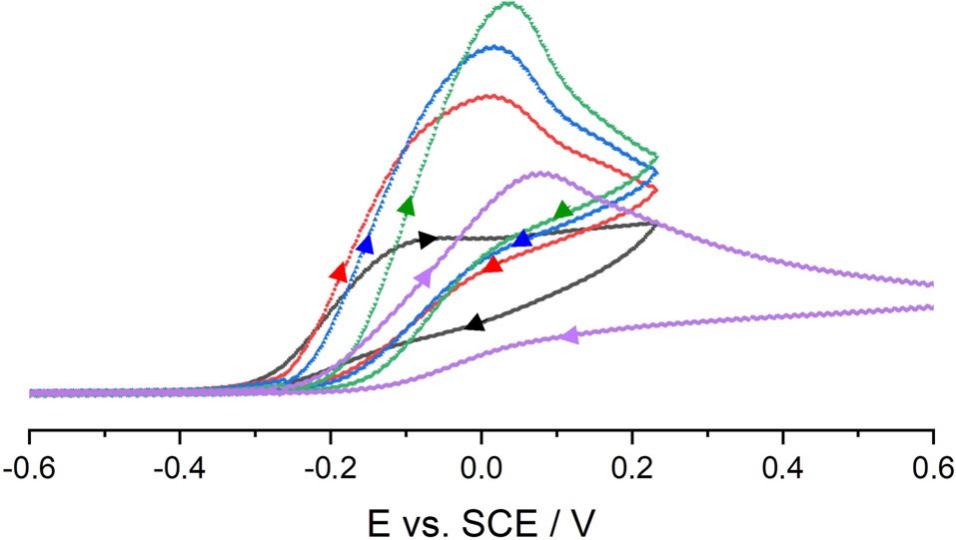
Cyclic voltammograms of **[1H][C_10_H_7_O]** (black), **[1H][(C_10_H_7_O)_2_H]** (purple) and **[1H][C_10_H_7_O]** with concentrations of 0.1 m (red), 0.2 m (blue) and 0.6 m H_2_O (green) in the electrolyte solution. Voltammograms recorded in 0.1 m [NBu_4_][PF_6_]THF solution at 100 mV s^−1^ under inert atmosphere with a glassy carbon working electrode (2.0(1) mm), a counter electrode (steel 18/8, 2.0(1) mm) and an Ag/AgCl reference electrode. All potentials were calibrated to the Fc/Fc^+^ couple (+0.405 V vs. SCE).

The investigation of analogous 2‐naphtholate anions deliver similar trends of the observed oxidation potentials as for [PhO]^−^ anions. The determined value of **[1H][C_10_H_7_O]** with *E*_Ox_=−0.15(1) V vs. SCE shifts significantly with formation of the hydrogen bond in **[1H][(C_10_H_7_O)_2_H]** (*E*_Ox_=+0.08(1) V vs. SCE, Figure 9). Also in this case, *E*_Ox_ of the non‐coordinated anion in [C_10_H_7_O]^−^ is clearly shifted compared to the literature data (+0.10 V vs. SCE).^[12]^ As expected, the subsequent addition of water to the **[1H][C_10_H_7_O]** electrolyte solution (0.1 m, 0.2 m, and 0.6 m H_2_O) leads to gradually shifts of *E*_Ox_ (+0.00(1) V, +0.01(1) V, +0.03(1) V vs. SCE, Figure 9). The picture gets completed by treatment of **[1H][(C_10_H_7_O)_2_H]** with an excess of H_2_O, which shifts the oxidation potential cathodically (*E*_Ox_=+0.06(1) V vs. SCE).

**Figure 10:**
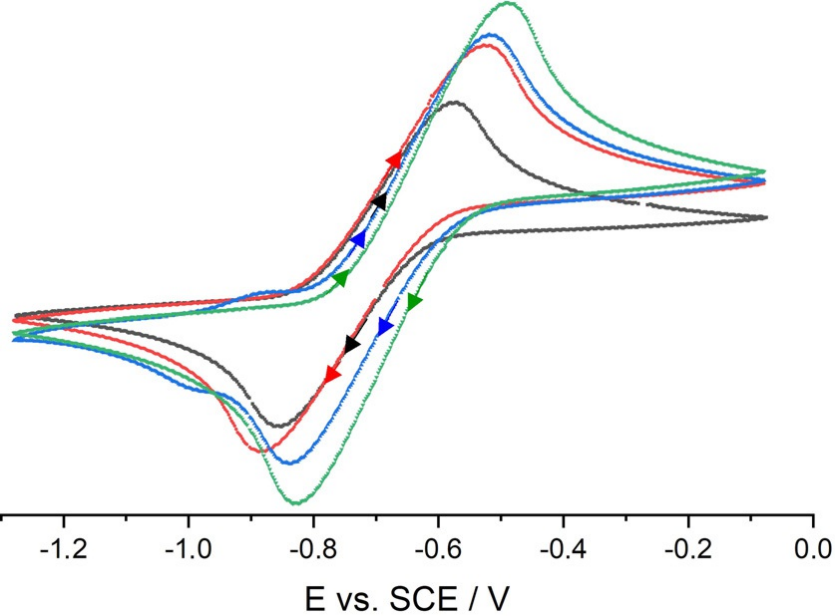
Cyclic voltammograms of **[1H][^MeO*t*Bu2^PhO]** (black), **[1H][(^MeO*t*Bu2^PhO)_2_H]** (red) and **[1H][(^MeO*t*Bu2^PhO)_2_H]** with concentrations of 0.1 m (blue) and 0.2 m H_2_O (green) in the electrolyte solution. Voltammograms recorded in 0.1 m [NBu_4_][PF_6_]THF solution at 100 mV s^−1^ under inert atmosphere with a glassy carbon working electrode (2.0(1) mm), a counter electrode (steel 18/8, 2.0(1) mm) and an Ag/AgCl reference electrode. All potentials were calibrated to the Fc/Fc^+^ couple (+0.405 V vs. SCE).

Interestingly, CV measurements of the hydrogen bonded adduct in **[1H][(^MeO*t*Bu2^PhO)_2_H]** reveal a similar redox potential (−0.70(1) V vs. SCE, Figure 10) as for the non‐coordinated phenolate [^MeO*t*Bu2^PhO]^−^ (−0.72(1) V vs. SCE).

In comparison to phenolate [PhO]^−^ and [(PhO)_2_H]^−^, the small redox shift between [^MeO*t*Bu2^PhO]^−^ and [(^MeO*t*Bu2^PhO)_2_H]^−^ may be rationalized by weaker hydrogen bonding in [(^MeO*t*Bu2^PhO)_2_H]^−^ relative to [(PhO)_2_H]^−^. This fact could possibly lead to a more pronounced dissociation of [(^MeO*t*Bu2^PhO)_2_H]^−^ in solution and could be responsible for the small redox shift between the coordinated and non‐coordinated anion. Subsequent addition of water to the **[1H][(^MeO*t*Bu2^PhO)_2_H]**‐electrolyte solution leads to further anodically shifted potentials of *E* ^0^=−0.68(1) V and −0.66(1) V vs. SCE (0.1 m and 0.2 m H_2_O). Likewise, the addition of water (0.05 m, 0.1 m, 0.4 m and 1.9 m) to **[1H][^MeO*t*Bu2^PhO]** in the electrolyte solution lowers the observed redox potentials (*E* ^0^=−0.71(1) V, −0.70(1) V, −0.66(1) V and −0.58(1) V vs. SCE).

### Phenolates for one‐electron reductions

Having non‐coordinated phenolate anions and their hydrogen bonded adducts in hand, the second part of this paper is focused on their reducing properties in SET reactions.

In general, radical anions are accessible by electrochemical reduction processes or by single‐electron transfer reactions. Especially organic representatives featuring conjugated π‐systems exhibit low lying π*‐orbitals and enable the formation of stable radical anions. The “*E. coli*”[^22^, ^23^] of electron transfer reagents is the well‐known electron‐acceptor tetracyanoethylene (TCNE),^[24]^ which is attracting considerable interest for applications in organic semiconductor materials^[25]^ or organic magnets.^[26]^

The reduction of tetracyanoethylene to its radical anion [TCNE]^.−^ is usually instrumented by the reaction with alkali or transition metals, like elemental potassium or copper, but can also be effected by potassium iodide.^[27]^ The incorporated metal cations may be replaced by other cations via salt metathesis reactions.^[28]^

**Scheme 4:**
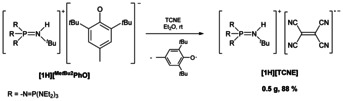
Synthesis of **[1H][TCNE]** by one electron reduction of TCNE applying phenolate salt **[1H][^Me*t*Bu2^PhO]**.

As discussed before, non‐coordinated phenolate anions as one electron transfer reagents should be of low nucleophilicity and the formed phenoxyl radicals should not undergo any further reactions. For this purpose, salt **[1H][^Me*t*Bu2^PhO]** is reacted with TCNE in ethereal solution (Scheme 4).

**Figure 11:**
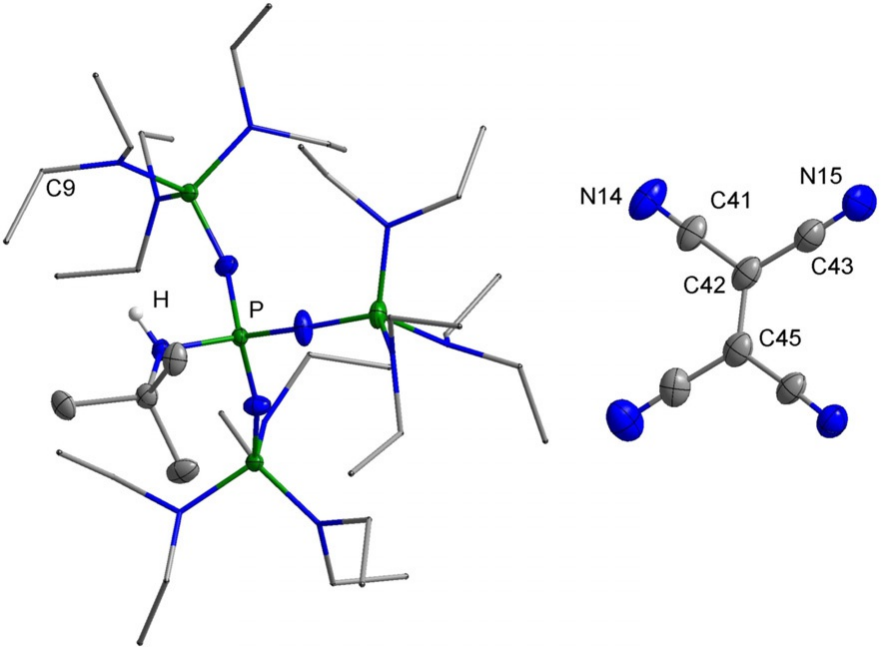
Molecular structure of the radical anion salt **[1H][TCNE]**. Thermal ellipsoids are shown at 50 % probability. The hydrogen atoms bonded at carbon and the minor occupied disordered N(C_2_H_5_)_2_ group are omitted for clarity. Diethylamino groups are shown as stick model. Selected bond lengths [pm] and angles [°]: C41−C42 141.5(1), C42−C43 142.8(1), C42−C45 141.7(1), N14−C41 114.4(1), N15−C43 114.6(1); C41‐C42‐C43 118.9(2), C41‐C42‐C45 120.7(2), C43‐C42‐C45 120.3(2).

A rapid electron transfer is observed, which is accompanied by an immediate color change from colorless to green‐yellow. Advantageously, the formed radical anion salt **[1H][TCNE]** precipitates from the reaction mixture as a deep‐orange solid in an 88 % yield. The phenoxyl radicals can be completely removed by extraction with diethyl ether. The formation of **[1H][TCNE]** is ascertained by elemental analysis and X‐ray investigation (Figure 11).

Although the formation of the dimeric [TCNE]_2_^2−^ dianion is known,^[29]^ the [TCNE]^.−^ radical anion in **[1H][TCNE]** is strictly monomeric. This anion is well separated from the counterion with the closest contact of N15‐C9 with 326.6(1) pm. The C42‐C45 bond in **[1H][TCNE]** of 141.7(1) pm well compares with that of calculated and isolated [TCNE]^.−^ radical anions.[^23^, ^28^, ^30^]

With the application of the “*E. coli*”[^22^, ^23^] of electron transfer reagents we confirmed the proof of concept for the preparation of radical anion salts with a weakly coordinating phosphazenium cation, which originates from phosphazenium phenolates.

In view of the severe environmental pollution caused by our economy, which is evident in the climate change on our planet, we tried to find further practical applications for the newly synthesized phosphazenium phenolates.

Sulfur hexafluoride is the strongest greenhouse gas presently known. Its extreme chemical inertness has a dramatic impact on our climate.^[31]^ Clearly, methods for the successful degradation of sulfur hexafluoride are urgently required. Numerous papers are addressing SF_6_ activation with transition metal complexes of titanium,^[32]^ rhodium,^[33]^ platinum,^[34]^ chromium and vanadium,^[35]^ as well as of nickel.^[36]^ In all cases the principal reactions lead to corresponding sulfido and fluoride metal complexes. The activation of SF_6_ can also be performed electrochemically^[37]^ or by single‐electron transfer reactions, as demonstrated by the reaction of SF_6_ with alkali metals in liquid ammonia.^[38]^ SET reactions of SF_6_ with organic electron donors,[^39^, ^40^] TEMPOLi^[41]^ and also photo‐activated systems^[42]^ have been described. The mechanism for the SF_6_ degradation is not completely understood. While some papers claim that the activation proceeds via an SET prior to the disintegration of the corresponding [SF_6_]^⋅−^ radical anion,[^39^, ^41^, ^43^] Dielmann et al. postulate a nucleophilic activation with the use of highly electron rich phosphanes.^[44]^

**Scheme 5:**
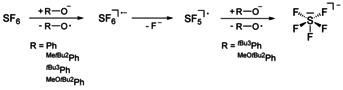
Activation of SF_6_ with phosphazenium phenolate salts.

In this context, the reducing properties of all synthesized non‐coordinated phenolate anions were tested for the activation of SF_6_ (Scheme 5).

As previously reported,^[10]^ treatment of the strongest reducing reagent **[1H][^MeO*t*Bu2^PhO]** with SF_6_ in ethereal solution leads to the spontaneous formation of the pentafluorosulfanide anion ([SF_5_]^−^) and a color change from yellow to deep red, for which the corresponding liberated phenoxyl radicals seem responsible.

The formation of the pentafluorosulfanide anion from SF_6_ is reported to proceed via two single‐electron transfer steps (Scheme 5).[^39^, ^41^] The intermediately formed radical anion [SF_6_]^.−^ of the first reduction step disintegrates into F^−^ and an (SF_5_)^.^ radical, and the latter is reduced by a second phenolate to obtain the [SF_5_]^−^ anion.

The reaction of F^−^ with the borosilicate glass surface leads to the formation of several fluorides, mainly [HF_2_]^−^, as evidenced by ^19^F NMR spectroscopy. Storage of the collected red ethereal ^MeO*t*Bu2^PhO^.^ phenoxyl radical solution at −28 °C and most likely diffusion of water and oxygen into the solution afforded green crystals of the corresponding decomposition product 2,6‐di‐*tert*‐butylbenzoquinone, which was authenticated by single‐crystal X‐ray diffraction. The reaction of the ammonium salt **[NBu_4_][^MeO*t*Bu2^PhO]** with sulfur hexafluoride also leads to the formation of [SF_5_]^−^. However, the rate of the reaction seems significantly decreased compared to its phosphazenium analogue, and the anion [SF_5_]^−^ was detected not before three days of reaction time.

The treatment of SF_6_ with tri‐*tert*‐butyl phenolate **[1H][** ^***t*****Bu3**^ **PhO]** in THF as well enables the formation of the [SF_5_]^−^ anion and fluorides (mainly [HF_2_]^−^), as evidenced by ^19^F NMR spectroscopy. The characteristic resonances of the pseudo square‐pyramidal [SF_5_]^−^ anion[^10^, ^45^, ^46^] are observed in the ^19^F NMR spectrum as a quintet at *δ*=88.9 ppm and a doublet at 59.5 ppm, both showing the characteristic ^2^ *J*_FF_ coupling of 45 Hz.^[45]^ The colorless reaction solution turns deep blue over time, which well agrees with the color of the free phenoxyl radical.^[46]^ ^19^F NMR spectroscopic investigations manifest the stability of the [SF_5_]^−^ anion in solution in the presence of phenoxyl radicals over weeks of storage at ambient temperature.

The phenolates **[1H][^Me*t*Bu2^PhO]** and **[1H][PhO]** are also utilized for SF_6_ activation. However, in sharp contrast to the sterically encumbered 2,4,6‐tri‐*tert*‐butyl‐ and 2,6‐di‐*tert*‐butyl‐4‐methoxy‐ substituted phenolates, the reactions of **[1H][^Me*t*Bu2^PhO]** and **[1H][PhO]** with SF_6_ did not lead to the formation of the pentafluorosulfanide anion, but only afforded fluorides. Since [^Me*t*Bu2^PhO]^−^ and [PhO]^−^ show a chemical irreversibility in CV experiments, this observation may be rationalized by the reaction of phenoxyl radicals with intermediates prior to the generation of the [SF_5_]^−^ anion. The hydrogen bonded anionic adduct in **[1H][(^MeO*t*Bu2^PhO)_2_H]** was also reacted with SF_6_. After stirring of the reaction mixture over three days, the solution turned red and the formation of [SF_5_]^−^ was monitored by ^19^F NMR spectroscopy. In contrast to the reaction with the non‐coordinated anion [^MeO*t*Bu2^PhO]^−^, the rate of the formation of [SF_5_]^−^ by [(^MeO*t*Bu2^PhO)_2_H]^−^ was also significantly decreased. Presumably by the presence of phenolic OH functions, several unspecified fluorine containing products were further formed and detected by ^19^F NMR spectroscopy.

Interestingly, the reducing agent tetrakis(dimethylamino)ethylene (TDAE) is reported to be not capable for the SF_6_ activation,^[39]^ although TDAE is an even stronger reducing agent (*E* ^0^=−0.78 V vs. SCE) than all presented phenolate anions herein. This suggests the conclusion that the redox strength itself is not the only factor for a successful reduction of sulfur hexafluoride. In accordance with Dielmann et al.^[44]^ one possible explanation for the success of the SF_6_ activation with phenolates invokes a nucleophilic interaction of a phenolate anion with a fluorine atom of SF_6_, which may support the subsequent activation by single‐electron transfer.

## Conclusions

We succeeded in high‐yield syntheses of a series of non‐coordinated phenolate anions by deprotonation of the corresponding alcohols with the tetraphosphazene base **1**. The phenolate anions show significantly shortened C−O^−^ bonds (128.4(2) pm to 129.8(2) pm) compared to coordinated phenolate anions like in NaOPh (133(1) pm). With phosphazene **1** hydrogen bonded phenol‐phenolate and phenolate hydrates are accessible by the deprotonation of phenol in the presence of one equivalent of phenol or water, respectively. This renders the investigation of the influence of hydrogen bonding on the redox potentials of phenolate anions possible. The latter were studied by cyclic voltammetric measurements and reveal significant shifts of the oxidation potentials of [PhO]^−^ (−0.12(1) V vs. SCE) by contact to a water molecule (−0.04(1) V vs. SCE) or to a phenol molecule (+0.22(1) V vs. SCE). The same trend was observed by comparison of the non‐coordinated 2‐naphtholate salt **[1H][C_10_H_7_O]** (−0.15(1) V) with the 2‐naphthol adduct in **[1H][(C_10_H_7_O)_2_H]** (+0.08(1) V). The latter anion displays the strongest observed hydrogen bond within all presented phenol‐phenolates with an O‐O separation of 238.5(4) pm, which results in a hypsochromic shift of the absorption into the UV light relative to [C_10_H_7_O]^−^, which displays two absorption bands in the visible light (412 and 439 nm). The strong hydrogen bond is also perceptible in fluorescence emission spectra. The non‐coordinated anion displays a single fluorescence maximum at *λ*_em_=462 nm (*λ*_ex_=320 nm, Stokes shift 9605 cm^−1^). In contrast, the fluorescence of the adduct is of less intensity and exhibits several maxima at *λ*_em_=344, 360, 427 and 458 nm, respectively. The hydrogen bond in the phenol‐phenolate adduct **[1H][(^MeO*t*Bu2^PhO)_2_H]** featuring bulky *tert*‐butyl substituents in 2 and 6 position is slightly elongated (O−O distance 247.0(1) pm) compared to the non‐substituted analogue [(PhO)_2_H]^−^ (243.7(2) pm). Consequently, the redox potential of the free phenolate [^MeO*t*Bu2^PhO]^−^ (−0.72(1) V vs. SCE) is only minor influenced by hydrogen bonding to the phenol with a difference of 20 mV.

We also disclosed the potential of non‐coordinated phenolates as one‐electron reducing agents. By application of tetracyanoethylene as the “*E. coli*”[^22^, ^23^] of electron transfer reagents, we presented the possibility for the preparation of radical anion salts from phosphazenium phenolates and isolated the corresponding salt **[1H][TCNE]** in high yield (88 %). We further described the reduction of the chemically inert sulfur hexafluoride with phosphazenium phenolates, which in case of the sterically encumbered phenolates [^MeO*t*Bu2^PhO]^−^ and [^*t*Bu3^PhO]^−^ resulted in the formation of pentafluorosulfanide anions. The reduction with other phenolates merely gave fluorides.

## Experimental Section

**Materials, instrumentation, methods**: All chemicals were obtained from commercial sources and used without further purification. All solvents were carefully dried and freshly distilled prior to use. Standard high‐vacuum techniques were employed throughout all experiments. Non‐volatile compounds were handled in a dry N_2_ atmosphere using Schlenk techniques. Syntheses of phosphazene **1**,^[16]^ phenolates **[1H][PhO]**, **[1H][^MeO*t*Bu2^PhO]** and **[1H][(PhO)_2_H]** and the reaction of **[1H][^MeO*t*Bu2^PhO]** with SF_6_ were performed according to literature procedures.^[10]^

NMR spectra were recorded on a Bruker Avance III 500 spectrometer (^1^H 500.01 MHz; ^13^C 125.73 MHz; ^19^F 470.48 MHz; ^31^P 202.41 MHz) or on a Bruker Avance III 500 HD spectrometer (^1^H 500.20 MHz; ^13^C 125.78 MHz; ^19^F 470.66 MHz; ^31^P 202.48 MHz). Positive shifts are downfield from the external standards TMS (^1^H, ^13^C), CCl_3_F (^19^F) and H_3_PO_4_ (^31^P). The NMR spectra were recorded in the indicated deuterated solvent or in relation to [D_6_]acetone‐filled capillaries.

IR spectra were recorded on an ALPHA‐FT‐IR spectrometer (Bruker) using an ATR unit with a diamond crystal for liquids and solids.

Elemental analyses were performed by Mikroanalytisches Laboratorium Kolbe (Oberhausen, Germany). The elemental analyses of **[1H][(^MeO*t*Bu2^PhO)_2_H]**, **[1H][PhO(H_2_O)]** were performed in the element‐analytical laboratory of the Universität Bielefeld using the EURO EA Element Analyzer 2010 (HEKAtech GmbH).

Melting points were measured on a Mettler Toledo Mp70 Melting Point System.

The UV/Vis spectroscopic investigations were performed using the UV/Vis‐spectroscopy‐system 8453 (Agilent) with a closable cuvette (*d*=1 cm) containing a stirring bar (8 mm) under inert atmosphere at 20 °C. The cuvette was heated to 100 °C for 30 minutes prior to each measurement. All samples were prepared in flame‐dried Schlenk flasks with concentrations of about 32 μm in THF, which was carefully dried over K and freshly distilled prior to use.

The fluorescence emission spectra were recorded on a RF‐5301PC (Shimadzu) in a quartz glass cuvette (*d*=1 cm) applying substance concentrations of 200 μm in THF. All samples were prepared in flame‐dried Schlenk flasks using THF, which was carefully dried over K and freshly distilled prior to use. The samples were excited with a Xenon lamp at *λ*_ex_=320 nm.

The cyclic voltammetric investigations were performed on a PGSTAT101 potentiostat (Metrohm) using a “three‐electrode arrangement” in a flame‐dried 25 mL Schlenk flask under inert atmosphere with a glassy carbon working electrode (2.0(1) mm diameter), a counter electrode (stainless steel 18/8, 2.0(1) mm diameter) and an Ag/AgCl reference electrode in a saturated ethanolic LiCl solution (148 mV vs. SHE). The supporting electrolyte [NBu_4_][PF_6_] was carefully dried in a high vacuum (10^−3^ mbar). THF was dried over K and freshly distilled prior to use. For every run 0.1 mmol of the substrate and 15 mL of the electrolyte solution were used. The Fc/Fc^+^ couple was used as internal standard by adding a small amount (spatula tip) of ferrocene after the measurements. The obtained redox potentials were finally recalculated based on the Fc/Fc^+^ couple which was set at *E* ^0^(Fc/Fc^+^)= +0.405 V vs. SCE. In case of **[1H][PhO]** and **[1H][C_10_H_7_O]** the addition of ferrocene leads to changes in the observed oxidation potentials, and therefore *E* ^0^(Fc/Fc^+^)=+0.673 V vs. Ag/AgCl was used as the external reference for the recalculation vs. SCE.

Nano‐ESI mass spectra were recorded using an Esquire 3000 ion trap mass spectrometer (Bruker Daltonik GmbH, Bremen, Germany) equipped with a nano‐ESI source. Samples were dissolved in THF and introduced to static nano‐ESI using in‐house pulled glass emitters. Nitrogen served both as nebulizer gas and dry gas. Nitrogen was generated by a Bruker nitrogen generator NGM 11. Helium served as cooling gas for the ion trap and collision gas for mass spectrometry experiments. The mass axis was externally calibrated with ESI‐L Tuning Mix (Agilent Technologies, Santa Clara, CA, USA) as calibration standard.

The crystal data were collected on a Rigaku Supernova diffractometer (Cu‐K_α_ radiation (*λ*=154.184 pm) or Mo‐K_α_ radiation (*λ*=71.073 pm) at 100.0(2) K. Using Olex2,^[47]^ the structures were solved with the ShelXT^[48]^ structure solution program using direct methods and refined with the ShelXL^[49]^ refinement package using least squares minimization. All hydrogen atoms bonded at nitrogen or oxygen were refined isotropically including the 1:1 disordered ones in **[1H][(PhO)_2_H]**. Details of the X‐ray investigation are given in Tables 2 and 3.

**Table 2:** Structure refinement data of **[1H][PhO(H_2_O)]**, **[1H][** ^***t*****Bu3**^ **PhO]**, **[NBu_4_][^MeO*t*Bu2^PhO]** and **[1H][(^MeO*t*Bu2^PhO)_2_H]**.

Compound	**[1H][PhO(H_2_O)]**	**[1H][** ^***t*****Bu3**^ **PhO]**	**[NBu_4_][^MeO*t*Bu2^PhO]**	**[1H][(^MeO*t*Bu2^PhO)_2_H]**
empirical formula	C_46_H_107_N_13_O_2_P_4_	C_58_H_129_N_13_OP_4_	C_31_H_59_NO_2_	C_82_H_177_N_13_O_7_P_4_
a [pm]	1270.911(14)	1570.493(16)	919.264(10)	4964.04(4)
*b* [pm]	1334.105(14)	1952.776(17)	1726.121(16)	4964.04(4)
*c* [pm]	1727.395(19)	2297.32(2)	3817.73(3)	2016.72(2)
α [°]	81.8859(9)	90	90	90
β [°]	85.2605(9)	103.5089(11)	90	90
γ [°]	79.5752(9)	90	90	120
*V* [10^6^ pm^3^]	2846.76(5)	6850.55(12)	6057.82(10)	43 037.5(8)
Z	2	4	8	18
*ρ*_calc_ [mg mm^−3^]	1.165	1.114	1.048	1.098
crystal system	triclinic	monoclinic	orthorhombic	trigonal
space group	*P* $\bar{1}$	*P*2_1_/*c*	*P*2_1_2_1_2_1_	*R* $\bar{3}$
crystal size [mm^−3^]	0.332×0.258×0.096	0.301×0.093×0.067	0.339×0.142×0.12	0.424×0.381×0.247
μ [mm^−1^]	0.180	1.365	0.476	1.145
F(000)	1100.0	2544.0	2144.0	15 768.0
2*θ* range for data col. [°]	5.36 to 72.636	5.788 to 153.198	4.63 to 153.718	6.986 to 154.76
index ranges	−21≤*h*≤21 −22≤*k*≤22 −28≤*l*≤28	−19≤*h*≤19 −24≤*k*≤21 −28≤*l*≤28	−11≤*h*≤11 −21≤*k*≤21 −48≤*l*≤47	−50≤*h*≤61 −59≤*k*≤65 −24≤*l*≤25
reflections col.	27 5615	60 331	12 3549	14 9772
independent refl.	27 575	14 264	12 696	19 943
R(int)	0.0610	0.0418	0.0605	0.0490
data/restraints/parameter	27575/0/1027	14264/0/719	12696/0/1087	19943/0/1263
goodness‐of‐fit on F^2^	1.041	1.017	1.037	1.044
*R*_1_/w*R*_2_ [*I*>2*σ*(*I*)]	0.0377/0.0915	0.0369/0.0931	0.0311/0.0768	0.0382/0.0990
*R*_1_/w*R*_2_ (all data)	0.0559/0.1005	0.0426/0.0969	0.0336/0.0786	0.0401/0.1006
Δ*ρ*_max/min_ [e Å^−3^]	0.59/−0.43	0.54/−0.42	0.21/−0.16	0.91/−0.42
remarks	The water molecule is disordered with a ratio of 1:1		Inversion twin with a ratio of 1:1	A solvent mask was calculated, 2154 electrons were found, this is consistent with the presence of three molecules of diethyl ether per formula unit, which count 2268 electrons.
CCDC number	2035834	2035835	2035836	2035837

**Table 3:** Structure refinement data of **[1H][C_10_H_7_O]**, **[1H][(C_10_H_7_O)_2_H]** and **[1H][TCNE]**.

Compound	**[1H][C_10_H_7_O]**	**[1H][(C_10_H_7_O)_2_H]**	**[1H][TCNE]**
empirical formula	C_50_H_107_N_13_OP_4_	C_60_H_115_N_13_O_2_P_4_	C_46_H_100_N_17_P_4_
a [pm]	1321.29(3)	1625.06(3)	1111.084(6)
*b* [pm]	1413.71(3)	1804.57(3)	2734.353(13)
*c* [pm]	1630.93(3)	2300.42(4)	1927.356(13)
α [°]	94.8840(18)	90	90
β [°]	95.9651(17)	97.1688(15)	97.9077(6)
γ [°]	105.357(2)	90	90
*V* [10^6^ pm^3^]	2901.55(12)	6693.34(19)	5799.81(6)
Z	2	4	4
*ρ*_calc_ [mg mm^−3^]	1.179	1.166	1.163
crystal system	triclinic	monoclinic	monoclinic
space group	*P* $\bar{1}$	*P*2_1_/*n*	*P*2_1_/*n*
color shape			
crystal size [mm^−3^]	0.241×0.146×0.05	0.353×0.269×0.112	0.427×0.237×0.23
*μ* [mm^−1^]	0.177	0.163	1.563
F(000)	1132.0	2568.0	2220.0
2*θ* range for data col. [°]	5.222 to 72.964	5.174 to 72.638	5.646 to 153.29
index ranges	−21≤*h*≤22 −22≤*k*≤23 −27≤*l*≤26	−27≤*h*≤27 −30≤*k*≤30 −38≤*l*≤38	−13≤*h*≤13 −34≤*k*≤34 −23≤*l*≤23
reflections col.	91248	35 5625	10 4415
independent refl.	26 937	32 422	12 103
*R*(int)	0.0347	0.0783	0.0650
data/restraints/parameter	26937/0/1123	32422/0/1236	12103/7/655
goodness‐of‐fit on F^2^	1.048	1.031	1.047
*R*_1_/w*R*_2_ [*I*>2*σ*(*I*)]	0.0417/0.0951	0.0438/0.1030	0.0380/0.0993
*R*_1_/w*R*_2_ (all data)	0.0629/0.1040	0.0695/0.1151	0.0420/0.1027
Δ*ρ*_max/min_ [e Å^−3^]	0.89/−0.63	0.54/−0.38	0.53/−0.46
remarks	The anion and some ethyl groups are disordered with a ratio of 90:10, one ethyl group is disordered in a ratio of 59:41. Disordered atoms close together were constrained to have equivalent thermal parameters.	One naphtol molecule is disordered with a ratio of 70:30. The hydrogen atom is disordered between the two oxygen atoms with a ratio of 1:1. Disordered atoms close together were constrained to have equivalent thermal parameters	Disorder of one ‐N(Me)_2_ group over two sites (57:43). Bond lengths within this disorder were restraint to be equal.
CCDC number	2045902	2045903	2035838

Deposition numbers 2035834, 2035835, 2035836, 2035837, 2035838, 2045902, and 2045903 contain the supplementary crystallographic data for this paper. These data are provided free of charge by the joint Cambridge Crystallographic Data Centre and Fachinformationszentrum Karlsruhe Access Structures service www.ccdc.cam.ac.uk/structures.

**Synthesis of [1H][^Me*t*Bu2^PhO]**: Phosphazene **1** (4.17 g, 4.70 mmol) is dissolved in 20 mL of diethyl ether and the solution of 2,6‐di‐*tert*‐butyl‐4‐methylphenol (^Me*t*Bu2^PhOH, 1.04 g, 4.71 mmol) in 6 mL of diethyl ether is rapidly added. After 15 min a colorless solid separates. The suspension is stirred for additional 3 d (30 min are sufficient) and *n*‐hexane (10 mL) is added. The slight purple supernatant is removed via a syringe and the colorless solid is washed with additional 10 mL of *n*‐hexane. After drying in a high vacuum the product (5.11 g, 4.62 mmol, 98 %, based on 2,6‐di‐*tert*‐butyl‐4‐methylphenol) is isolated as a colorless solid (dec. >111 °C). Suitable crystals for XRD were grown from the ethereal reaction mixture at −28 °C. ^1^H NMR (500 MHz, [D_8_]THF, rt): *δ*=1.2 (t, ^3^ *J*_H,H_=7 Hz, 54 H, NCH_2_C**H**_3_), 1.4 (s, 9 H, NC(CH_3_)_3_), 1.4 (s, 18 H, C(CH_3_)_3_), 2.1 (s, 3 H, CH_3_), 2.2 (d, ^2^ *J*_P,H_=8 Hz, 1 H, NH), 3.2 (d, q, ^3^ *J*_P,H_=10 Hz, ^3^ *J*_H,H_=7 Hz, 36 H, NC**H**_2_CH_3_), 6.4 ppm (s, 2 H, *meta* H); ^13^C NMR (500 MHz, [D_8_]THF, rt): *δ*=12.9 (d, ^3^ *J*_P,C_=4 Hz, NCH_2_ **C**H_3_), 21.6 (s, CH_3_), 29.8 (s, C(**C**H_3_)_3_), 31.1 (d, ^3^ *J*_P,C_=5 Hz, NC(**C**H_3_)_3_), 34.8 (s, **C**(CH_3_)_3_), 39.1 (d, ^2^ *J*_P,C_=6 Hz, N**C**H_2_CH_3_), 50.5 (d, ^2^ *J*_P,C_=4 Hz, N**C**(CH_3_)_3_), 105.7 (s, *para* C), 122.9 (s, *ortho* C), 134.0 (s, *meta* C), 170.2 ppm (s, *ipso* C); ^31^P NMR (500 MHz, [D_8_]THF, rt): *δ*=−33.7 (q, d, ^2^ *J*_P,P_=70 Hz, ^2^ *J*_P,H_=8 Hz, 1 P, P=NH), 7.7 ppm (d, tridec, ^2^ *J*_P,P_=70 Hz, ^3^ *J*_P,H_=10 Hz, 3 P, (Et_2_N)_3_P); IR (ATR): $\tilde{\nu }$ =2964 (w), 2932 (w), 2870 (w), 2160 (vw), 1973 (vw), 1595 (vw), 1465 (w), 1420 (w), 1377 (m), 1350 (w), 1276 (s), 1226 (w), 1201 (s), 1173 (vs.), 1107 (w), 1074 (w), 1054 (w), 1017 (vs.), 942 (s), 887 (w), 848 (w), 793 (s), 741 (w), 700 (s), 612 (m), 508 (vs.), 448 (s), 437 (s), 399 (m), 379 (m) cm^−1^; MS (ESI, pos.) {*m*/*z* (%) [assignment]}: 886.7 (100) **[1H]^+^**; MS (ESI, neg.) {*m*/*z* (%) [assignment]}: 219.1 (100) **[^Me*t*Bu2^PhO]^−^**; elemental analysis calcd (%) for C_55_H_123_N_13_OP_4_: C 59.70, H 11.20, N 16.46; found: C 59.74, H 11.13, N 16.34.

**Synthesis of [1H][** ^***t*****Bu3**^ **PhO]**: Phosphazene **1** (4.86 g, 5.49 mmol) is dissolved in 20 mL of diethyl ether and the solution of 2,4,6‐tri‐*tert*‐butylphenol (^*t*Bu3^PhOH, 1.44 g, 5.50 mmol) in 5 mL of diethyl ether is rapidly added. Immediately a colorless solid precipitates from the slightly yellow mixture. The suspension is stirred for additional 3 d (30 min are sufficient) and *n*‐hexane (10 mL) is added. The slightly yellow supernatant is removed via a syringe and the solid is washed with additional 10 mL of *n*‐hexane. After drying in a high vacuum the product (6.24 g, 5.43 mmol, 99 %, based on ^*t*Bu3^PhOH) is isolated as a colorless solid (dec. >196 °C). Suitable crystals for XRD were grown from the ethereal reaction mixture at −28 °C. ^1^H NMR (500 MHz, [D_8_]THF, rt): *δ*=1.2 (t, ^3^ *J*_H,H_=7 Hz, 54 H, NCH_2_C**H**_3_), 1.2 (s, 9 H, *para* C(CH_3_)_3_), 1.4 (s, 9 H, NC(CH_3_)_3_), 1.5 (s, 18 H, *ortho* C(CH_3_)_3_), 2.2 (d, ^2^ *J*_P,H_=8 Hz, 1 H, NH), 3.3 (d, q, ^3^ *J*_P,H_=10 Hz, ^3^ *J*_H,H_=7 Hz, 36 H, NC**H**_2_CH_3_), 6.7 ppm (s, 2 H, *meta* H); ^13^C NMR (500 MHz, [D_8_]THF, rt): *δ*=13.0 (d, ^3^ *J*_P,C_=4 Hz, NCH_2_ **C**H_3_), 29.9 (s, *ortho* C(**C**H_3_)_3_), 31.1 (d, ^3^ *J*_P,C_=5 Hz, NC(**C**H_3_)_3_), 32.4 (s, *para* C(**C**H_3_)_3_), 33.5 (s, *para* **C**(CH_3_)_3_), 35.3 (s, *ortho* **C**(CH_3_)_3_), 39.1 (d, ^2^ *J*_P,C_=6 Hz, N**C**H_2_CH_3_), 50.1 (d, ^2^ *J*_P,C_=4 Hz, N**C**(CH_3_)_3_), 118.4 (s, *ortho* C), 119.5 (s, *para* C), 133.1 (s, *meta* C), 170.3 ppm (s, *ipso* C); ^31^P NMR (500 MHz, [D_8_]THF, rt): *δ*=−33.7 (q, d, ^2^ *J*_P,P_=70 Hz, ^2^ *J*_P,H_=8 Hz, 1 P, P=NH), 7.7 ppm (d, tridec, ^2^ *J*_P,P_=70 Hz, ^3^ *J*_P,H_=10 Hz, 3 P, (Et_2_N)_3_P); IR (ATR): $\tilde{\nu }$ =2965 (vw), 2934 (w), 2870 (w), 1466 (w), 1425 (w), 1375 (m), 1352 (w), 1307 (s), 1290 (vs.), 1239 (w), 1223 (w), 1203 (s), 1176 (vs.), 1105 (w), 1073 (vw), 1055 (w), 1019 (vs.), 1001 (m), 943 (s), 920 (m), 887 (w), 869 (w), 845 (w), 828 (vw), 793 (s), 779 (m), 737 (w), 702 (s), 609 (m), 510 (vs.), 484 (s), 433 (s), 401 (w) cm^−1^; MS (ESI, pos.) {*m*/*z* (%) [assignment]}: 886.5 (100) **[1H]^+^**; MS (ESI, neg.) {*m*/*z* (%) [assignment]}: 261.1 (100) **[** ^***t*****Bu3**^ **PhO]^−^**; elemental analysis calcd (%) for C_58_H_129_N_13_OP_4_: C 60.65, H 11.32, N 15.85; found: C 60.76, H 11.46, N 15.73.

**Synthesis of [NBu_4_][^MeO*t*Bu2^PhO]**: Tetra‐*n‐butyl*ammonium hydroxide triacontahydrate (641 mg, 0.80 mmol) is suspended in diethyl ether (10 mL) and THF (2 mL) and the solution of 2,6‐di‐*tert*‐butyl‐4‐methoxyphenol (^MeO*t*Bu2^PhOH, 189 mg, 0.80 mmol) in 5 mL of diethyl ether is rapidly added to yield a yellow suspension. After stirring for two hours the solvent is removed under reduced pressure and the product is dried in a high vacuum overnight. The product (338 mg, 0.72 mmol, 90 % based on ^MeO*t*Bu2^PhOH) is isolated as a pale yellow crystalline solid (dec. >77 °C). The product slowly decomposes upon air contact, which results in a color change to green. Suitable crystals for XRD were grown from a saturated THF/Et_2_O solution at −28 °C. ^1^H NMR (500 MHz, [D_8_]THF, rt): *δ*=1.0 (t, ^3^ *J*_H,H_=7 Hz, 12 H, NCH_2_CH_2_CH_2_C**H**_3_), 1.4 (m, 8 H, NCH_2_CH_2_C**H**_2_CH_3_), 1.5 (s, 18 H, C(CH_3_)_3_), 1.8 (m, 8 H, NCH_2_C**H**_2_CH_2_CH_3_), 3.5 (m, 8 H, NC**H**_2_CH_2_CH_2_CH_3_), 3.6 (s, 3 H, OCH_3_), 6.5 ppm (s, 2 H, *meta* H); ^13^C NMR (500 MHz, [D_8_]THF, rt): *δ*=13.2 (s, NCH_2_CH_2_CH_2_ **C**H_3_), 19.7 (s, NCH_2_CH_2_ **C**H_2_CH_3_), 24.1 (s, NCH_2_ **C**H_2_CH_2_CH_3_), 29.7 (s, C(**C**H_3_)_3_), 35.0 (s, **C**(CH_3_)_3_), 57.0 (s, OCH_3_), 58.7 (s, N**C**H_2_CH_2_CH_2_CH_3_), 111.0 (s, *meta* C), 135.0 (s, *ortho* C), 143.3 (s, *para* C), 164.0 ppm (s, *ipso* C); IR (ATR): $\tilde{\nu }$ =2941 (w), 2895 (w), 2873 (w), 2821 (vw), 1488 (w), 1461 (m), 1410 (vs.), 1376 (w), 1366 (m), 1349 (w), 1312 (w), 1282 (w), 1248 (w), 1210 (m), 1196 (w), 1161 (w), 1150 (vw), 1103 (vw), 1066 (vs.), 1029 (vw), 924 (w), 890 (w), 853 (w), 811 (vw), 788 (w), 779 (s), 750 (w), 739 (w), 685 (vw), 650 (vw), 603 (vw), 572 (vw), 562 (vw), 538 (vw), 521 (vw), 496 (vw), 470 (vw), 461 (vw), 438 (w), 406 (vw), 383 (w) cm^−1^; MS (ESI, pos.) {*m*/*z* (%) [assignment]}: 242.2 (100) **[NBu_4_]^+^**; MS (ESI, neg.) {*m*/*z* (%) [assignment]}: 235.1 (48) **[^MeO*t*Bu2^PhO]^−^**.

**Synthesis of [1H][C_10_H_7_O]**: Phosphazene **1** (774 mg, 0.87 mmol) is dissolved in diethyl ether (15 mL) and the solution of 2‐naphthol (C_10_H_7_OH, 126 mg, 0.87 mmol) in 5 mL of diethyl ether is rapidly added to yield a green‐yellow fluorescent solution from which a solid precipitates. The suspension is stirred for 5 minutes and then cooled to −28 °C overnight. The supernatant solution is removed via a syringe and the product (887 mg, 0.86 mmol, 99 % based on **1**) is isolated after drying in a high vacuum as a fine fluorescent green crystalline solid (dec. >107 °C). Exposure to air results in a fast discoloration. Suitable crystals for XRD were grown from the ethereal reaction mixture at −28 °C. ^1^H NMR (500 MHz, [D_8_]THF, rt): *δ*=1.2 (t, ^3^ *J*_H,H_=7 Hz, 54 H, NCH_2_C**H**_3_), 1.4 (s, 9 H, NC(CH_3_)_3_), 2.3 (d, ^2^ *J*_P,H_=8 Hz, 1 H, NH), 3.2 (d, q, ^3^ *J*_P,H_=10 Hz, ^3^ *J*_H,H_=7 Hz, 36 H, NC**H**_2_CH_3_), 6.1 (s, 1 H, aryl H), 6.3 (m, 1 H, aryl H), 6.5 (m, 1 H, aryl H), 6.7 (m, 1 H, aryl H), 7.0 (m, 1 H, aryl H), 7.1 (m, 1 H, aryl H), 7.1 ppm (m, 1 H, aryl H); ^13^C NMR (500 MHz, [D_8_]THF, rt): *δ*=13.0 (d, ^3^ *J*_P,C_=4 Hz, NCH_2_ **C**H_3_), 31.1 (d, ^3^ *J*_P,C_=5 Hz, NC(**C**H_3_)_3_), 39.1 (d, ^2^ *J*_P,C_=6 Hz, N**C**H_2_CH_3_), 50.5 (d, ^2^ *J*_P,C_=4 Hz, N**C**(CH_3_)_3_), 107.5 (aryl C), 111.7 (aryl C), 122.0 (aryl C), 122.3 (aryl C), 126.3 (aryl C), 126.6 (aryl C), 129.0 (aryl C), 139.3 (aryl C), 173.7 ppm (C‐O^−^); ^31^P NMR (500 MHz, [D_8_]THF, rt): *δ*=−33.7 (q, d, ^2^ *J*_P,P_=70 Hz, ^2^ *J*_P,H_=7 Hz, 1 P, P=NH), 7.7 ppm (d, tridec, ^2^ *J*_P,P_=70 Hz, ^3^ *J*_P,H_=10 Hz, 3 P, (Et_2_N)_3_P); IR (ATR): $\tilde{\nu }$ =2967 (vw), 2931 (vw), 2868 (vw), 1602 (vw), 1585 (vw), 1543 (vw), 1490 (vw), 1462 (w), 1433 (w), 1375 (w), 1353 (w), 1278 (s), 1254 (s), 1227 (m), 1202 (s), 1172 (vs.), 1104 (m), 1054 (w), 1019 (vs.), 941 (vs.), 844 (w), 832 (m), 789 (m), 756 (w), 734 (m), 721 (w), 705 (s), 693 (s), 625 (w), 614 (m), 593 (w), 524 (s), 508 (vs.), 467 (vs.), 455 (s), 431 (s), 418 (s), 496 (m), 386 (m) cm^−1^; MS (ESI, pos.) {*m*/*z* (%) [assignment]}: 886.8 (100) **[1H]^+^**; MS (ESI, neg.) {*m*/*z* (%) [assignment]}: 143.0 (100) **[C_10_H_7_O]^−^**, 287.1 (5) **[(C_10_H_7_O)_2_H]^−^**; elemental analysis calcd for C_50_H_107_N_13_OP_4_: C 58.28, H 10.47, N 17.67; found: C 58.32, H 10.76, N 17.03.

**Synthesis of [1H][(C_10_H_7_O)_2_H]**: Phosphazene **1** (714 mg, 0.81 mmol) is dissolved in diethyl ether (10 mL) and the solution of 2‐naphthol (C_10_H_7_OH, 232 mg, 1.61 mmol) in 5 mL of diethyl ether is rapidly added to yield a strong green‐blue fluorescent solution. The fluorescence disappears after the complete addition of 2‐naphthol and a second light green phase forms. The emulsion is stirred for 5 min and is then cooled to −28 °C overnight. Since no precipitation of the desired product occurs, the upper phase is removed via a syringe and the lower phase is evaporated to dryness. After drying in a high vacuum the product (733 mg, 0.62 mmol, 77 % based on **1**) is isolated as a colorless crystalline solid (m.p. 76 °C). Suitable crystals for XRD were grown from a concentrated ethereal solution at −28 °C. ^1^H NMR (500 MHz, [D_8_]THF, rt): *δ*=1.1 (t, ^3^ *J*_H,H_=7 Hz, 54 H, NCH_2_C**H**_3_), 1.3 (s, 9 H, NC(CH_3_)_3_), 2.1 (d, ^2^ *J*_P,H_=8 Hz, 1 H, NH), 3.2 (d, q, ^3^ *J*_P,H_=10 Hz, ^3^ *J*_H,H_=7 Hz, 36 H, NC**H**_2_CH_3_), 6.8 (m, 2 H, aryl H), 7.0 (m, 2 H, aryl H), 7.0 (m, 2 H, aryl H), 7.2 (m, 2 H, aryl H), 7.4 (m, 4 H, aryl H), 7.5 ppm (m, 2 H, aryl H); ^13^C NMR (500 MHz, [D_8_]THF, rt): *δ*=12.9 (d, ^3^ *J*_P,C_=4 Hz, NCH_2_ **C**H_3_), 31.0 (d, ^3^ *J*_P,C_=5 Hz, NC(**C**H_3_)_3_), 39.0 (d, ^2^ *J*_P,C_=6 Hz, N**C**H_2_CH_3_), 50.4 (d, ^2^ *J*_P,C_=4 Hz, N**C**(CH_3_)_3_), 108.6 (aryl C), 117.7 (aryl C), 123.5 (aryl C), 124.7 (aryl C), 125.6 (aryl C), 126.9 (aryl C), 127.1 (aryl C), 137.0 (aryl C), 165.1 ppm (C‐O); ^31^P NMR (500 MHz, [D_8_]THF, rt): *δ*=−33.7 (q, d, ^2^ *J*_P,P_=70 Hz, ^2^ *J*_P,H_=7 Hz, 1 P, P=NH), 7.7 ppm (d, tridec, ^2^ *J*_P,P_=70 Hz, ^3^ *J*_P,H_=10 Hz, 3 P, (Et_2_N)_3_P); IR (ATR): $\tilde{\nu }$ =3379 (vw), 3044 (vw), 2970 (w), 2929 (vw), 2870 (vw), 1621 (vw), 1597 (vw), 1561 (vw), 1499 (vw), 1465 (vw), 1453 (w), 1417 (w), 1378 (m), 1349 (w), 1312 (m), 1295 (m), 1249 (s), 1226 (m), 1202 (s), 1171 (vs.), 1112 (w), 1074 (w), 1055 (w), 1017 (vs.), 941 (s), 865 (w), 842 (s), 789 (s), 742 (vs.), 699 (vs.), 610 (s), 536 (s), 515 (vs.), 471 (s), 453 (s), 427 (s) cm^−1^; MS (ESI, pos.) {*m*/*z* (%) [assignment]}: 886.9 (100) **[1H]^+^**; MS (ESI, neg.) {*m*/*z* (%) [assignment]}: 143.1 (100) **[C_10_H_7_O]^−^**, 287.1 (7) **[(C_10_H_7_O)_2_H]^−^**; elemental analysis calcd for C_60_H_115_N_13_O_2_P_4_: C 61.36, H 9.87, N 15.50; found: C 61.32, H 9.78, N 15.05.

**Synthesis of [1H][(^MeO*t*Bu2^PhO)_2_H]**: Phosphazene **1** (1.31 g, 1.47 mmol) is dissolved in diethyl ether (10 mL) and the solution of 2,6‐di‐*tert*‐butyl‐4‐methoxyphenol (^MeO*t*Bu2^PhOH, 699 mg, 2.96 mmol) in 5 mL of diethyl ether is rapidly added to yield a deep green‐yellow solution. The solution is stirred for 5 minutes and then cooled to −28 °C overnight by which green crystals precipitate. The supernatant solution is removed via a syringe and the product (2.00 g, 1.47 mmol, 100 % based on **1**) is isolated after drying in a high vacuum as a fine green crystalline powder (m.p. (dec.) >74 °C). Exposure to air results in a slow decomposition accompanied by a color change from green to brown‐green. Suitable crystals for XRD were grown from the ethereal reaction mixture at −28 °C. ^1^H NMR (500 MHz, [D_8_]THF, rt): *δ*=1.2 (t, ^3^ *J*_H,H_=7 Hz, 54 H, NCH_2_C**H**_3_), 1.4 (s, 9 H, NC(CH_3_)_3_), 1.4 (s, 36 H, C(CH_3_)_3_), 2.2 (d, ^2^ *J*_P,H_=8 Hz, 1 H, NH), 3.2 (d, q, ^3^ *J*_P,H_=10 Hz, ^3^ *J*_H,H_=7 Hz, 36 H, NC**H**_2_CH_3_), 3.6 (s, 6 H, OCH_3_), 6.5 (s, 4 H, *meta* H), 10.5 ppm (s, br, OH); ^13^C NMR (500 MHz, [D_8_]THF, rt): *δ*=12.9 (d, ^3^ *J*_P,C_=4 Hz, NCH_2_ **C**H_3_), 30.3 (s, C(**C**H_3_)_3_), 31.1 (d, ^3^ *J*_P,C_=5 Hz, NC(**C**H_3_)_3_), 35.0 (s, **C**(CH_3_)_3_), 39.1 (d, ^2^ *J*_P,C_=6 Hz, N**C**H_2_CH_3_), 50.5 (d, ^2^ *J*_P,C_=4 Hz, N**C**(CH_3_)_3_), 55.5 (s, OCH_3_), 109.8 (s, *meta* C), 138.5 (s, *ortho* C), 147.6 (s, *para* C), 158.5 ppm (s, *ipso* C); ^31^P NMR (500 MHz, [D_8_]THF, rt): *δ*=−33.7 (q, d, ^2^ *J*_P,P_=70 Hz, ^2^ *J*_P,H_=7 Hz, 1 P, P=NH), 7.7 ppm (d, tridec, ^2^ *J*_P,P_=70 Hz, ^3^ *J*_P,H_=10 Hz, 3 P, (Et_2_N)_3_P); IR (ATR): $\tilde{\nu }$ =2961 (w), 2934 (w), 2869 (w), 1461 (w), 1441 (vw), 1411 (w), 1376 (m), 1352 (m), 1279 (s), 1223 (w), 1203 (s), 1176 (vs.), 1108 (w), 1056 (m), 1019 (vs.), 942 (vs.), 891 (w), 848 (m), 817 (w), 791 (s), 779 (vs.), 740 (m), 703 (vs.), 682 (m), 643 (m), 608 (s), 587 (m), 537 (vs.), 525 (s), 509 (vs.), 479 (s), 463 (vs.), 453 (vs.), 438 (vs.), 405 (s), 390 (s) cm^−1^; MS (ESI, pos.) {*m*/*z* (%) [assignment]}: 886.9 (100) **[1H]^+^**; MS (ESI, neg.) {*m*/*z* (%) [assignment]}: 235.1 (85) **[^MeO*t*Bu2^PhO]^−^**; elemental analysis calcd for C_70_H_147_N_13_O_4_P_4_: C 61.87, H 10.90, N 13.40; found: C 61.69, H 11.67, N 12.83.

**Synthesis of [1H][PhO(H_2_O)]**: Phosphazene **1** (579 mg, 0.65 mmol) is dissolved in 7 mL of diethyl ether and the solution of phenol (61 mg, 0.65 mmol) in 2 mL of diethyl ether is rapidly added. Immediately a second pale yellow phase forms. Subsequently water (15 mg, 0.83 mmol) is added and the emulsion is stirred for an additional hour prior to cooling to −28 °C. The supernatant is removed from the colorless crystalline solid via a syringe and the solid is dried in a low vacuum (down to 10 mbar). The product (618 mg, 0.62 mmol, 95 %, based on phenol) is isolated as a colorless, slight hygroscopic, sticky crystalline solid (dec. >92 °C). Suitable crystals for XRD were grown from the ethereal reaction mixture at −28 °C. ^1^H NMR (500 MHz, [D_8_]THF, rt): *δ*=1.2 (t, ^3^ *J*_H,H_=7 Hz, 54 H, NCH_2_C**H**_3_), 1.4 (s, 9 H, NC(CH_3_)_3_), 2.2 (d, ^2^ *J*_P,H_=8 Hz, 1 H, NH), 3.2 (d, q, ^3^ *J*_P,H_=10 Hz, ^3^ *J*_H,H_=7 Hz, 36 H, NC**H**_2_CH_3_), 4.3 (s, br, 2 H, H_2_O), 5.7 (s, 1 H, *para* H), 6.2 (s, 2 H, *ortho* H), 6.6 ppm (m, 2 H, *meta* H); ^13^C NMR (500 MHz, [D_8_]THF, rt): *δ*=13.0 (d, ^3^ *J*_P,C_=4 Hz, NCH_2_ **C**H_3_), 31.1 (d, ^3^ *J*_P,C_=5 Hz, NC(**C**H_3_)_3_), 39.1 (d, ^2^ *J*_P,C_=6 Hz, N**C**H_2_CH_3_), 50.5 (d, ^2^ *J*_P,C_=4 Hz, N**C**(CH_3_)_3_), 104.5 (s, *para* C), 119.0 (s, *ortho* C), 127.6 (s, *meta* C), 173.6 ppm (s, *ipso* C); ^31^P NMR (500 MHz, [D_8_]THF, rt): *δ*=−33.7 (q, d, ^2^ *J*_P,P_=70 Hz, ^2^ *J*_P,H_=8 Hz, 1 P, P=NH), 7.7 ppm (d, tridec, ^2^ *J*_P P_=70 Hz, ^3^ *J*_P H_=10 Hz, 3 P, (Et_2_N)_3_P); IR (ATR): $\tilde{\nu }$ =3350 (vw), 3047 (vw), 2967 (w), 2930 (vw), 2867 (w), 1577 (w), 1538 (vw), 1484 (w), 1460 (w), 1415 (vw), 1377 (w), 1350 (w), 1326 (w), 1259 (s), 1227 (w), 1201 (s), 1175 (vs.), 1108 (w), 1054 (w), 1018 (vs.), 979 (m), 942 (vs.), 848 (m), 821 (w), 795 (m), 740 (m), 699 (s), 691 (s), 614 (w), 590 (w), 513 (vs.), 486 (s), 457 (s), 435 (s) cm^−1^; MS (ESI, pos.) {*m*/*z* (%) [assignment]}: 886.9 (100) **[1H]^+^**; MS (ESI, neg.) {*m*/*z* (%) [assignment]}: 92.9 (100) **[PhO]^−^**; elemental analysis calcd for C_46_H_107_N_13_O_2_P_4_: C 55.34, H 10.80, N 18.24; found: C 54.88, H 10.77, N 17.28.

**Synthesis of [1H][TCNE]**: The salt **[1H][^Me*t*Bu2^PhO]** (522 mg, 0.47 mmol) is suspended in 12 mL of diethyl ether and a solution of tetracyanoethylene (64 mg, 0.50 mmol) in 10 mL of diethyl ether is rapidly added. Immediately a deep green‐yellow suspension forms. The suspension is stirred for 5 minutes and then cooled to −28 °C overnight. The supernatant is removed via a syringe, the solid is washed with diethyl ether (2×10 mL) and dried in a high vacuum. The product (422 mg, 0.42 mmol, 88 %, based on **[1H][^Me*t*Bu2^PhO]**) is isolated as an orange solid (dec. >86 °C). Suitable crystals for XRD were grown from the ethereal reaction mixture at −28 °C. IR (ATR): $\tilde{\nu }$ =2968 (vw), 2932 (vw), 2869 (vw), 2182 (vw), 2143 (w), 2123 (vw), 1465 (vw), 1414 (vw), 1378 (w), 1350 (w), 1267 (m, br), 1225 (w), 1202 (s), 1175 (vs.), 1108 (w), 1054 (w), 1018 (vs.), 942 (vs.), 920 (m), 845 (w), 794 (s), 738 (w), 699 (s), 612 (m), 510 (vs.), 442 (s), 408 (m) cm^−1^; MS (ESI, pos.) {*m*/*z* (%) [assignment]}: 886.6 (100) **[1H]^+^**; MS (ESI, neg.) {*m*/*z* (%) [assignment]}: 127.8 (37) [TCNE]^⋅−^; HRMS (ESI, pos.) *m*/*z* calcd for C_40_H_100_N_13_P_4_: 886.71696; found: 886.7177; HRMS (ESI, neg.) *m*/*z* calcd for C_6_N_4_: calcd: 128.01284; found: 128.0128; elemental analysis calcd for C_46_H_100_N_17_P_4_: C 54.42, H 9.93, N 23.45; found: C 55.57, H 10.11, N 22.65.

**Activation of SF6**: *General procedure* : The phosphazenium phenolate salt (60 mg) is filled into a Young NMR tube containing an [D_6_]acetone filled capillary. First THF or Et_2_O (0.4 mL) as the solvent is condensed onto the salt at −196 °C prior to the condensation of an excess of SF_6_ (3 mbar, 0.075 mmol) onto the mixture. The reaction is allowed to warm to ambient temperature and the course of the reaction is monitored by ^19^F NMR spectroscopy.

## Conflict of interest

The authors declare no conflict of interest.
